# Appraisal of Antiophidic Potential of Marine Sponges against *Bothrops jararaca* and *Lachesis muta* Venom

**DOI:** 10.3390/toxins5101799

**Published:** 2013-10-17

**Authors:** Camila Nunes Faioli, Thaisa Francielle Souza Domingos, Eduardo Coriolano de Oliveira, Eládio Flores Sanchez, Suzi Ribeiro, Guilherme Muricy, Andre Lopes Fuly

**Affiliations:** 1 Department of Molecular and Cellular Biology, Institute of Biology, Federal Fluminense University, Niteroi 24020-141, RJ, Brazil; E-Mails: camilafaioli@hotmail.com (C.N.F.); thaisadomingos@yahoo.com.br (T.F.S.D.); eduardocoriolano@globo.com (E.C.O.); 2 Department of Marine Biology, Institute of Biology, Federal Fluminense University, Niteroi 24020-141, RJ, Brazil; 3 Ezequiel Dias Foundation, Belo Horizonte 30510-010, MG, Brazil; E-Mail: eladio@funed.mg.gov.br; 4 Department of Invertebrates, Federal University of Rio de Janeiro, National Museum 20940-040, RJ, Brazil; E-Mails: suzimr@yahoo.com.br (S.R.); muricy@mn.ufrj.br (G.M.)

**Keywords:** Brazilian sponges, *Bothrops jararaca*, *Lachesis muta*, snake venom, neutralization

## Abstract

Snakebites are a health problem in many countries due to the high incidence of such accidents. Antivenom treatment has regularly been used for more than a century, however, this does not neutralize tissue damage and may even increase the severity and morbidity of accidents. Thus, it has been relevant to search for new strategies to improve antiserum therapy, and a variety of molecules from natural sources with antiophidian properties have been reported. In this paper, we analyzed the ability of ten extracts from marine sponges (*Amphimedon viridis*, *Aplysina fulva*, *Chondrosia collectrix*, *Desmapsamma anchorata*, *Dysidea etheria*, *Hymeniacidon heliophila*, *Mycale angulosa*, *Petromica citrina*, *Polymastia janeirensis*, and *Tedania ignis*) to inhibit the effects caused by *Bothrops jararaca* and *Lachesis muta* venom. All sponge extracts inhibited proteolysis and hemolysis induced by both snake venoms, except *H*. *heliophila*, which failed to inhibit any biological activity. *P*. *citrina* inhibited lethality, hemorrhage, plasma clotting, and hemolysis induced by *B*. *jararaca* or *L*. *muta*. Moreover, other sponges inhibited hemorrhage induced only by *B*. *jararaca*. We conclude that Brazilian sponges may be a useful aid in the treatment of snakebites caused by *L*. *muta* and *B*. *jararaca* and therefore have potential for the discovery of molecules with antiophidian properties.

## 1. Introduction

Snakebite envenomation is an important public health problem in tropical, subtropical, and developing countries, and according to World Health Organization is a neglected disease [1]. Snake bite is estimated to affect more than five million people per year, with 100,000 death and around three times as many amputations or other disabilities; although the exact number of is unknown [1,2]. Snake venoms are composed of a complex mixture of enzymes and non-enzymatic proteins that interact and interfere with several systems of the body, affecting haemostasis and leading to pathophysiological disturbances. Envenomation by snakes causes systemic effects as well as local reactions in the region of the bite [3,4,5]. Systemic effects include neurotoxicity, cardiovascular, renal and respiratory symptoms, hemolysis, and hemorrhage; while locally there may be tissue necrosis causing permanent disability resulting in limb amputation, hemorrhage, edema, pain, and inflammatory reactions [6,7,8]. In South America, *B*. *jararaca* has a higher incidence of accidents (95%) than *L*. *muta* (2%), although *L*. *muta* bites lead to more severe symptoms and have a lethality index three times higher than *B*. *jararaca* [9].

Until now, snake antivenoms have been the only effective treatment able to counteract most of the symptoms that follow snakebites [1,9,10,11]. However, antivenoms have some disadvantages, as they may produce side effects (from mild fever to anaphylactic reactions), poorly inhibit local effects, and have high production costs [1,12,13,14]. The production of higher quality, safer, and cheaper antivenoms is a challenge worldwide. For this, it is important to search for molecules capable of neutralizing local effects more efficiently, and to use them together with antivenoms to complement the effectiveness of serum treatment. 

The seas provide an amazing source of molecules when compared to the terrestrial environment. Among 34 phyla, 17 occur on land and 32 occur in the sea. Marine organisms produce molecules with a chemical diversity derived from primary (such as polypeptides, enzymes, and polysaccharides) or secondary (including terpenes, alkaloids and sterols) metabolisms, with a variety of pharmacological and ecological functions [15,16]. Over 60% of bioactive compounds are from marine fauna of which 70% come from sponges [17,18,19,20]. Sponges (phylum Porifera) are the most primitive of multicellular animals. They have existed for 700–800 million years occurring primarily in marine environments at different depths [21]. There are about 8000 species of sponges described which are divided into three groups: Calcarea (five orders, 24 families), Demospongiae (15 orders, 92 families), and Hexactinellida (six orders, 20 families), however, their true diversity may be higher [22,23]. Products isolated from sponges display antiviral [24], anticancer [25], and antimicrobial [26], effects on platelet aggregation [27] and on endothelial cells [28,29]. Some of these products resulted in the synthesis of Ara-A (anticancer) and Ara-C (antiviral) drugs, which were further approved for clinical use [30,31,32], although their marketing proved to be too expensive. Therefore, bioprospecting in sponges is potentially important for discovering new molecules with antivenom properties in this highly diverse group of animals.

In the present study, the effect has been evaluated of ten Brazilian marine sponge species (*A*. *viridis*, *A*. *fulva*, *C*. *collectrix*, *D*. *anchorata*, *D*. *etheria*, *H*. *heliophila*, *M*. *angulosa*, *P*. *citrina*, *P*. *janeirensis*, *T*. *ignis*) against *in vivo* (hemorrhage, edema, and lethality) and *in vitro* (hemolysis, proteolysis, and clotting) activities induced by *B*. *jararaca* and *L*. *muta* snake venom.

## 2. Results

### 2.1. Neutralization of Proteolysis

**Figure 1:**
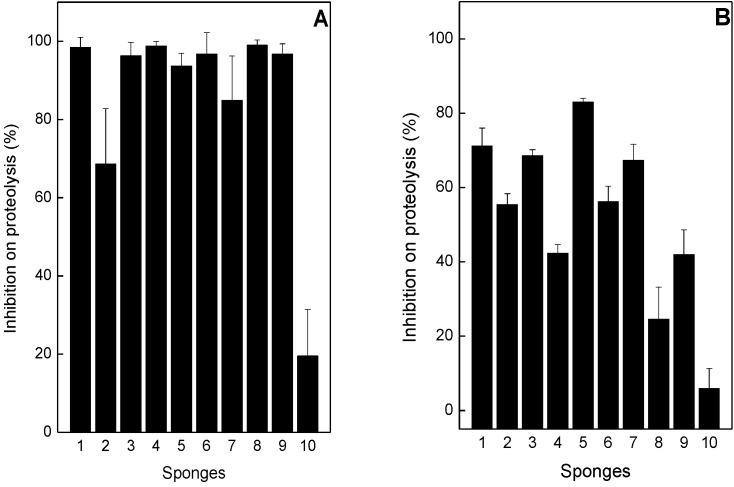
Effect of sponges’ extracts on proteolysis induced by *B*. *jararaca* or *L*. *muta* venom. Marine sponges (132 μg/mL) were incubated for 30 min at room temperature with 34 μg/mL *B*. *jararaca* (Panel **A**) or with 32 μg/mL *L*. *muta* (Panel **B**), and then proteolysis test performed. *M*. *angulosa* (column 1), *C*. *collectrix* (column 2), *T*. *ignis* (column 3), *A*. *fulva* (column 4), *D*. *etheria* (column 5), *D*. *anchorata* (column 6), *A*. *viridis* (column 7), *P*. *citrina* (column 8), *P*. *janeirensis* (column 9), *H*. *heliophila* (column 10). Data are means ± SE of two individual experiments (*n* = 3).

*B*. *jararaca* and *L*. *muta* venoms hydrolyzed azocasein in a concentration-dependent manner with one EC of 17 µg/mL and 16 µg/mL, respectively (data not shown). As shown in Figure 1, the sponge extracts (132 µg/mL) inhibited proteolysis induced by two EC of *B*. *jararaca* and *L*. *muta* venom with different potencies. As observed in the Figure 1A, at 1:8 (*w*/*w*) venom:sponge ratio, *M*. *angulosa* (column 1), *T*. *ignis* (column 3), *A*. *fulva* (column 4), *D*. *etheria* (column 5), *D*. *anchorata* (column 6), *P*. *citrina* (column 8), and *P*. *janeirensis* (column 9) inhibited 100% of proteolytic activity of *B*. *jararaca* venom, while *Chondrosia* sp. (column 2) and *A*. *viridis* (column 7) inhibited around 70%–80%. According to Figure 1B, at 1:8 (*w*/*w*) venom:sponge ratio, the maximum inhibition against *L*. *muta* venom was 80%, achieved by *D*. *etheria*. The sponge *H*. *heliophila* did not inhibit proteolytic activity induced by both venoms.

### 2.2. Neutralization of Hemorrhage

**Figure 2:**
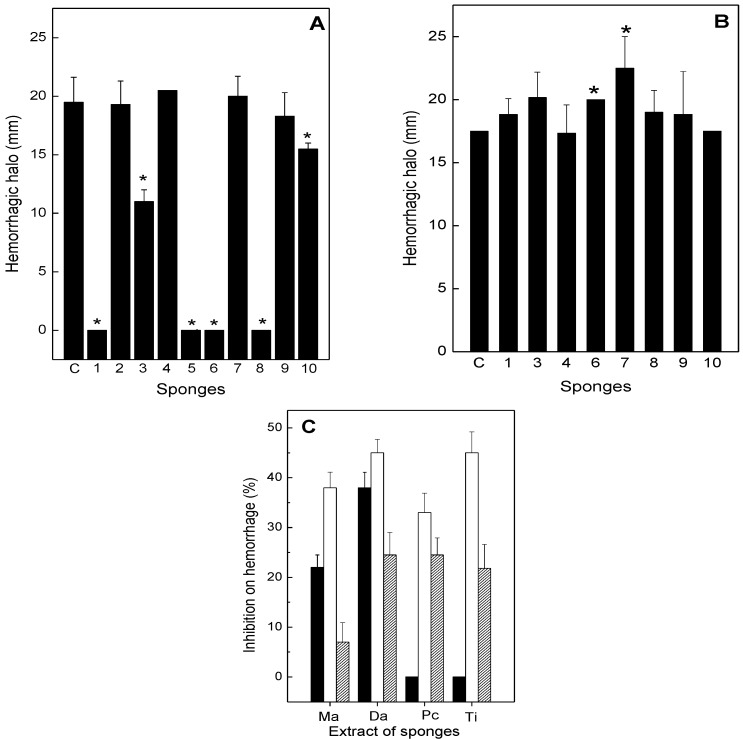
Effect of the sponge extracts on hemorrhage induced by *B*. *jararaca* or *L*. *muta* venom. The sponge extracts (220 µg/g) were incubated for 30 min. at room temperature with 20 µg/g *B*. *jararaca* (Panel **A**) or with 10 µg/g *L*. *muta* venom (Panel **B**), then the mixtures were injected into mice and the hemorrhage test results evaluated, as described in the methods. Columns are: Venom with DMSO (Column C), *M*. *angulosa* (column 1), *C*. *collectrix* (column 2), *T*. *ignis* (column 3), *A*. *fulva* (column 4), *D*. *etheria* (column 5), *D*. *anchorata* (column 6), *A*. *viridis* (column 7), *P*. *citrina* (column 8), *P*. *janeirensis* (column 9), *H*. *heliophila* (column 10). Data are expressed as means SEM of two individual experiments (*n* = 3). Panel **C**: *B*. *jararaca* venom (20 µg/g) was injected i.d., and 15 min. later, the sponge extracts *M*. *angulosa* (Ma), *D*. *anchorata* (Da), *P*. *citrine* (Pc) and *T*. *ignis* (Ti), 110 µg/g (black columns) or 220 µg/g (white columns) were injected i.d. or i.v. (220 µg/g, hatched columns). * Significance level (*p* < 0.05) when compared to columns C.

The effect of the sponge extracts on hemorrhagic activity of *B*. *jararaca* and *L*. *muta* venom was evaluated (Figure 2). Intradermal injection of 20 µg/g *B*. *jararaca* or 10 µg/g *L. muta* venom induced a hemorrhage halo in mice of 10 mm, which corresponds to one MHD. As shown in Figure 2A, *M*. *angulosa* (column 1), *D*. *etheria* (column 5), *D*. *anchorata* (column 6), and *P*. *citrina* (column 8), at a concentration of 220 µg/g (1:11 venom:sponge ratio, *w*/*w*), fully protected mice from hemorrhage caused by *B*. *jararaca* venom. However, when *Chondrosia* sp. (column 2), *A*. *fulva* (column 4), *D*. *etheria* (column 7) were incubated with *B*. *jararaca* venom, no protection was seen. The extracts of *T*. *ignis* (column 3) and *H*. *heliophila* (column 10) inhibited hemorrhage induced by *B*. *jararaca* around 40% and 20%, respectively (Figure 2A). In contrast, the extracts of sponges were not able to inhibit hemorrhage induced by *L*. *muta* venom (Figure 2B). Moreover, the sponge extracts (*M*. *angulosa*, *D*. *anchorata*, *P*. *citrine*, and *T*. *ignis*), at two concentrations (110 and 220 µg/g) were injected i.d. 15 min after *B*. *jararaca* venom (20 µg/g). In addition, at lower concentration, only the sponges *M*. *angulosa* and *D*. *anchorata* inhibited 20% and 40% the hemorrhagic activity of *B*. *jararaca* venom, respectively. However, at 220 µg/g, *P*. *citrine* (35%) and *T*. *ignis* (45%) inhibited *B-jararaca*-induced hemorrhage as well, and the percentage of inhibition of *M*. *angulosa* doubled (Figure 2C). In another injection protocol, *B*. *jararaca* venom (20 µg/g) was injected i.d. into mice, and the sponge extracts (220 µg/g) were administered intravenously 15 min later. Now, all the extracts inhibited hemorrhage, but with different profiles: *M*. *angulosa* (6%), *D*. *anchorata* (22%), *P*. *citrine* (22%) and *T*. *ignis* (20%) (Figure 2C).

### 2.3. Neutralization of Hemolysis

**Figure 3:**
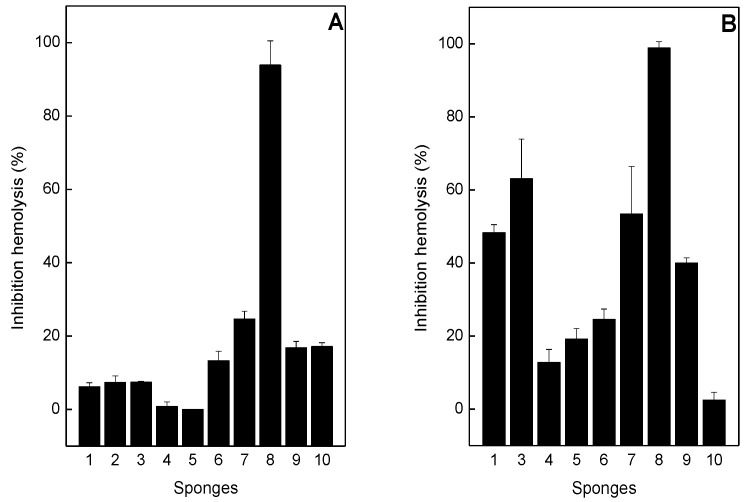
Effect of the sponge extracts on hemolysis induced by *B*. *jararaca* or *L*. *muta* venom. For Panel **A**, the sponge extracts (100 µg/mL) were incubated with 50 µg/mL *B*. *jararaca* and for Panel **B**, sponges (50 µg/mL) were incubated with 25 µg/mL *L*. *muta*, then hemolytic tests were performed. Columns are: Venoms incubated with *M*. *angulosa* (column 1), or with *C*. *collectrix* (column 2), or with *T*. *ignis* (column 3), or with *A*. *fulva* (column 4), or with *D*. *etheria* (column 5), or with *D*. *anchorata* (column 6), or with *A*. *viridis* (column 7), or with *P*. *citrina* (column 8), or with *P*. *janeirensis* (column 9), or with *H*. *heliophila* (column 10). Data are expressed as means SEM of three individual experiments (*n* = 3).

As shown in Figure 3A, most of the sponge extracts, at 1:2 venom:sponge ratio (*w*/*w*) inhibited hemolysis induced by *B*. *jararaca* from 0% to 20%, with the exception of *P*. *citrina* sponge, that inhibited 90% of hemolysis. Similar results were obtained with *P*. *citrina* for inhibition of *L*. *muta*-induced hemolysis (Figure 3B). However, most of the other sponges inhibited over 20% of hemolysis induced by *L*. *muta* venom (Figure 3B). The extracts of *A*. *fulva* (column 4), *D*. *etheria* (column 5) did not inhibit hemolysis induced by *B*. *jararaca* (Figure 3A) and *H*. *heliophila* (column 10) did not inhibit hemolysis induced by *L*. *muta* (Figure 3B).

### 2.4. Neutralization of coagulation

**Figure 4:**
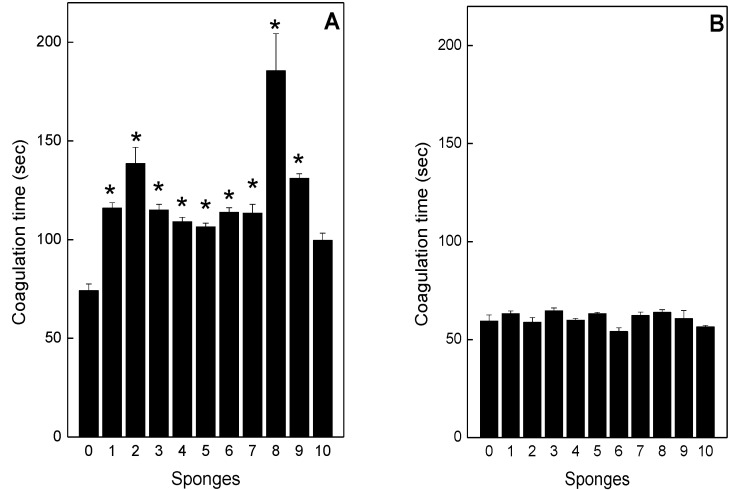
Effect of the sponge extracts on coagulation induced by *B*. *jararaca* or *L*. *muta*. *B*. *jararaca* (Panel **A**) or *L*. *muta* (Panel **B**) venoms were incubated with 0.5% DMSO (column 0), *M*. *angulosa* (column 1), *C*. *collectrix* (column 2), *T*. *ignis* (column 3), *A*. *fulva* (column 4), *D*. *etheria* (column 5), *D*. *anchorata* (column 6), *A*. *viridis* (column 7), *P*. *citrina* (column 8), *P*. *janeirensis* (column 9), *H*. *heliophila* (column 10), for 30 min at room temperature. After the mixture was added to the plasma, the clotting time was recorded, as described in the Methods. Data are expressed as means SEM of three individual experiments (*n* = 3). * Significance level (*p* < 0.05) when compared to column 0.

*B*. *jararaca* (50 μg/mL) and *L*. *muta* (10 µg/mL) venom clotted plasma after *ca*. 60 s and this was designated one MCD. When the venoms at 1 MCD were incubated with the sponge extracts, only inhibition of *B*. *jararaca*-induced clotting was observed (Figure 4A). Most sponges doubled the clotting time, when compared with the control value. However, *P*. *citrina* delayed clotting time approximately three times (Figure 4A, column 8). *H*. *heliophila* did not inhibit clotting induced by *B*. *jararaca* venom (Figure 4A, column 11). All sponge extracts failed to inhibit clotting induced by *L*. *muta* venom (Figure 4B). None of the sponge extracts induced clotting.

### 2.5. Neutralization of Edema and Lethality

**Figure 5:**
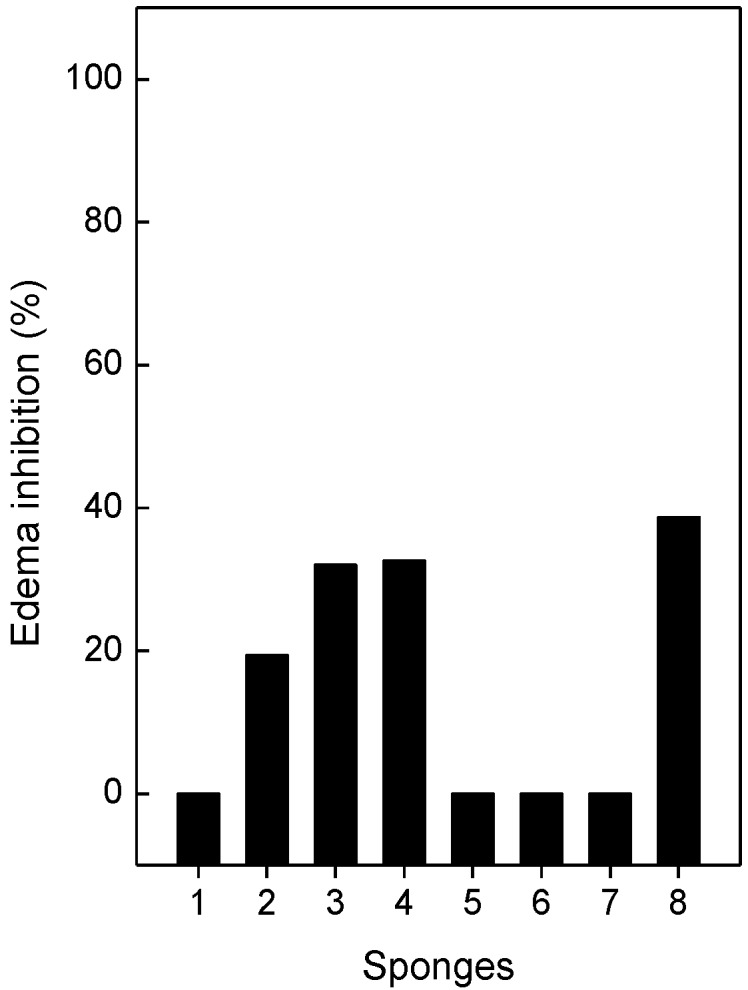
Effect of the sponge extracts on the edematogenic activity induced by *L*. *muta* venom. *L*. *muta* (0.4 µg/g) venom was incubated for 30 min at room temperature with 4.8 µg/g sponge extracts: *M*. *angulosa* (column 1), *T*. *ignis* (column 2), *A*. *fulva* (column3), *D*. *anchorata* (column 4), *A*. *viridis* (column 5), *P*. *citrina* (column 6), *P*. *janeirensis* (column 7), or *H*. *heliophila* (column 8). Data are expressed as means SEM of three individual experiments (*n* = 3).

A sub-plantar injection of *B*. *jararaca* (0.7 µg/g) or *L*. *muta* (0.4 µg/g) venom into the paws of mice induced an increase in paw volume of 26% and 44%, respectively. Subsequently, the sponge extracts were incubated with the venoms to give a final venom:ratio (*w*/*w*) of 1:14 for *B*. *jararaca* or 1:12 for *L*. *muta*, and the mixtures were injected into mice and edemas evaluated. None of the sponge extracts inhibited edema induced by *B*. *jararaca* venom, but different percentages of inhibition occurred for *L*. *muta*-induced edema (Figure 5). *T*. *ignis* (column 2) inhibited 20%, while *A*. *fulva* (column 3), *D*. *anchorata* (column 4), and *H*. *heliophila* (column 8), inhibited around 40% edema. When injected separately, the extracts did not induce edema in mice.

When *B*. *jararaca* venom (60 µg/g) was injected i.p. into mice, 100% lethality occurred after 126 min. (Table 1). The sponge extracts (12 µg/g) were incubated with *B*. *jararaca* venom to give a 1:2 venom:sponge ratio (*w*/*w*), and then mixtures were injected i.p. into mice. As shown in Table 1, 100% survival rates were observed for *A*. *fulva*, *A*. *viridis*, and *P*. *citrina* extracts up to 126 min. observation, with deaths only occurring at 258, 258, and 372 min, respectively. In contrast, the other sponge extracts protected the mice to a lesser extent. As only the sponge extracts of *A*. *fulva*, *A*. *viridis*, and *P*. *citrine* protected mice from death, another protocol of injection was done. The sponge extracts were injected either i.p. or i.v. 15 min after injection i.p. of *B*. *jararaca* venom. When sponges were injected i.p., no protection in mice was observed. However, a slight protection was seen for *A*. *fulva* when injection was performed i.v., in which mice that received only the venom died around 96 min and those treated with *A*. *fulva* died around 128 min (data not shown). None of the sponge extracts (500 µg/g) were lethal when injected i.p. into mice.

**Table 1:** Antilethality activity of the sponge extracts against *B*. *jararaca* venom.

Groups	Survival time (min)
*B*. *jararaca* + NaCl	126 ± 0.5
*B*. *jararaca* + DMSO	108 ± 0.9
*B*. *jararaca* + *M*. *angulosa*	150 ± 0
*B*. *jararaca* + *T*. *ignis*	180 ± 0
*B*. *jararaca* + *A*. *fulva*	258 ± 3.2
*B*. *jararaca* + *D*. *anchorata*	150 ± 0
*B*. *jararaca* + *A*. *viridis*	258 ± 3.2
*B*. *jararaca* + *P*. *citrine*	372 ± 3.2
*B*. *jararaca* + *P*. *janeirensis*	150 ± 0
*B*. *jararaca* + *H*. *heliophila*	168 ± 0.5

## 3. Discussion

In the present paper, it has been shown, for the first time, that extracts of marine sponges inhibit *in vivo* (hemorrhagic, edema and lethality) and *in vitro* (clotting, proteolytic and hemolytic) biological effects induced by snake venoms from *B*. *jararaca* and *L*. *muta*. However, the mechanism of inhibitory action of the sponges is not yet understood. Significant effects of *B*. *jararaca* and *L*. *muta* venoms, following snakebites have been reported previously [9,33]. Antivenom therapy not only fails to efficiently neutralize the local effects of envenomation, but also presents problems in its use [34,35]. The search for alternative treatments has therefore been intense and molecules from natural sources have been extensively prospected to result in discoveries such as a diterpene from the alga *Canistrocarpus cervicornis* [36]. However, to date, no effective bioproducts have been marketed as effective medicines for the treatment of envenomation by snakes. The plant kingdom has provided alternatives to antivenom therapy in the form of folk medicines to inhibit the symptoms of envenomation but such preparations are not widely accepted for use by the medical profession [37,38]. Very few previous reports have studied the potential of sponges in the treatment of envenomation [39,40,41].

Our results showed that sponge extracts inhibited the biological effects induced by *B*. *jararaca* and *L*. *muta* venoms, but with different potencies. These effects result from the action of proteases (serine and metalloproteases), which interfere with the blood-clotting cascade and blood vessels, which may lead to hemorrhage [3,4]. These enzymes in venoms are directly involved in the local effects that are responsible for amputations and deformities in victims as well as in the systemic symptoms. The injection of *B*. *jararaca* venom was lethal to mice after *ca*. two hours and only extracts of the sponges, *A*. *fulva*, *A*. *viridis*, or *P*. *citrine*, increased the survival rates of these mice. Other results with the sponge extracts were also promising, as both local and systemic actions of the venoms were also inhibited. The sponge extract inhibited hemolysis induced by the venoms and possibly this was due to a structural modification of phospholipase A_2_ (PLA_2_) enzymes or resulted from chelation of Ca^2+^ ions. PLA_2_s are another class of enzymes abundant in snake venoms with a vast variety of pathophysiological functions. These functions include erythrocyte lysis through a calcium-dependent reaction, myotoxicity, edema, hemorrhage, lethality, and effects on platelet aggregation [42,43]. Manoalide, a nonsteroidal sesterterpenoid isolated from the sponge *Luffariella* *variabilis* has already been shown to inhibit a cobra PLA_2_ by interacting with lysine residues in the enzyme [40].

Envenomation usually leads to intense inflammatory and edema reactions and PLA_2_ enzymes also participate together with other enzymes in these responses. Our results showed that the sponge extracts inhibited edema induced by *L*. *muta*, but not that resulting from *B*. *jararaca*. Extracts of *M*. *angulosa*, *D*. *etheria*, *D*. *anchorata*, and *P*. *citrina* inhibited *L*. *muta*-induced local hemorrhage, but not that caused by *B*. *jararaca*. Again, inhibition of edematogenic or hemorrhagic activity may have occurred by chelating divalent metals, such as Ca^2+^ or Zn^2+^, that are co-factors for enzymes involved both effects [39]. Despite similar composition of the *B*. *jararaca* and *L*. *muta* venoms, there are different isoforms that may explain the differences in the inhibitory profiles by the sponges. Analysis of phylogenetic trees shows that during evolutionary processes, some toxins lost or changed their activity and/or some key amino acid residues in the primary structure were replaced by others. This fact may lead to a diversity of physiological activities [44,45,46]. Among all the sponges tested, *P*. *citrina* inhibited all the biological activities induced by *L*. *muta* or *B*. *jararaca* venoms. Bastos *et al*. [47] have reported antiviral activity of *P*. *citrina*, but in fact its antiviral effect is due to the presence of symbiotic microorganisms. Most of sponges live in symbiosis with a diversity of microorganisms, representing more than 35% of the total body mass and they impart some of the biological functions to sponges, such as nutrition, defense and pharmacological properties [48,49]. However, some of the sponge bioactivities are due to the secondary metabolites synthesized during the sponge metabolism [50]. Finally, the different inhibitory profiles recorded by the sponge extracts against the effects of *B*. *jararaca* or *L*. *muta* venoms may have resulted from variations in the symbiotic microbial communities in the sponges.

## 4. Experimental Section

### 4.1. Venom and Animals

*Bothrops jararaca* and *Lachesis muta* snake venom were kindly supplied from the Fundação Ezequiel Dias (FUNED), Belo Horizonte, Minas Gerais state, Brazil, vacuum dried and stored at −20 °C until use. Balb/c mice (18–20 g) were obtained from the Núcleo de Animais de Laboratório (NAL) of the Universidade Federal Fluminense (UFF). They were housed under constant temperature (24 ± 1 °C) and light conditions. Experiments performed were approved by the UFF Institutional Committee for Ethics in Animal Experimentation (number 25) and were in accordance with the guidelines of the Brazilian Committee for Animal Experimentation (COBEA).

### 4.2. Marine Sponge Extracts

Specimens of marine sponges *M*. *angulosa*, *C*. *collectrix*, *T*. *ignis*, *A*. *fulva*, *D*. *anchorata*, *D*. *etheria*, *A*. *viridis*, *P*. *janeirensis*, *H*. *heliophila*, *P*. *citrina* were collected by free or scuba diving, extracted twice with acetone at room temperature (25 °C) for 24 h. Then, sponges were extracted for 72 h under the same conditions, filtered, and the solvent was evaporated off under reduced pressure, yielding a crude residue. An aliquot of each extract was weighed, aliquoted and frozen at −20 °C. The extracts of sponges were then dissolved in dimethyl sulfoxide (DMSO) to perform the biological assays. Voucher specimen of each sponge has been deposited at Museu Nacional of the Universidade Federal do Rio de Janeiro (MN/UFRJ), RJ, Brazil and authenticated by Dr. Guilherme Muricy and Dr. Suzi Ribeiro of the MN/UFRJ. The experimental procedures employed remove sugar and proteins, and extract less polar components and nonpolar ones, as diterpenes, alkaloids, fatty acids, and sterols.

### 4.3. Antiproteolytic Activity

Proteolytic activity of *B*. *jararaca* and *L*. *muta* venom was determined using azocasein as the substrate (0.2% *w*/*v*, in 20 mM Tris-HCl, 8 mM CaCl_2_, pH 8.8), with minor modifications [51]. An Effective Concentration (EC) was defined as the amount of venom (µg/mL) able to produce a variation of about 0.2 OD units at A 420. The inhibitory effect of sponges was performed by incubating them with two EC of *B*. *jararaca* and *L*. *muta* venom for 30 min at room temperature and then, proteolysis was measured. Control experiments were conducted by mixing venom with DMSO or saline and any proteolysis was recorded.

### 4.4. Antihemorrhagic Activity

Hemorrhagic lesions produced by *B*. *jararaca* and *L*. *muta* venom were quantified using a procedure described by Kondo [52], with minor modifications. Briefly, samples (100 µL) were injected intradermally (i.d.) into abdominal skin of mice. Two hours later, the animals were euthanized, abdominal skin removed, stretched, and inspected for visual changes in the internal aspect in order to localize hemorrhagic spots. Hemorrhage was quantified as the Minimum Hemorrhagic Dose (MHD), defined as the amount of venom (mg/kg) able to produce a hemorrhagic halo of 10 mm. The inhibitory effect of sponge extracts was investigated using three different protocols: (1) incubation samples with one MHD of *B*. *jararaca* and *L*. *muta* venom for 30 min at room temperature and then, the mixture (100 µL) was injected into mice and the hemorrhage was measured. For the other two protocols, one MHD of *B*. *jararaca* venom was injected i.d., and 15 min later, (2) the sponge extracts were injected i.d. at the same site where venom had been administered or 3) they were injected i.v. Hemorrhagic activity was expressed as the mean diameter (in millimeters) of the hemorrhagic halo induced by *B*. *jararaca* and *L*. *muta* venom in the absence and presence of the sponges. Negative control experiments were performed by injecting DMSO or saline.

### 4.5. Antihemolytic Activity

The degree of hemolysis of *B*. *jararaca* and *L*. *muta* venom was determined by the indirect hemolytic test using human erythrocytes and hen’s egg yolk emulsion as substrates [53]. The amount of *B*. *jararaca* and *L*. *muta* venom (µg/mL) that produced 100% hemolysis was denoted as Minimum Indirect Hemolytic Dose (MIHD). Inhibitory experiments were performed by incubating sponge extracts with one MIHD for 30 min at room temperature, and then hemolytic activity was evaluated. Control experiments were performed by incubating venom with DMSO or saline, instead of sponges.

### 4.6. Anticlotting Activity

The clotting activity of *B*. *jararaca* and *L*. *muta* venom was determined on an Amelung coagulometer, model KC4A (Labcon, Germany). Different concentrations of both venoms were mixed with a pool of human citrated plasma diluted in saline (1:1) from healthy volunteers from the local blood bank (Hospital Universitário Antônio Pedro—HUAP, Universidade Federal Fluminense). The amount of venom (µg/mL) that clots plasma in 60 seconds was denoted as the Minimum Coagulant Dose (MCD). To evaluate the inhibitory effect, sponge extracts were preincubated for 30 min at room temperature with one MCD of venom, and then, the mixture was added to plasma and clotting time recorded. Control experiments were performed in parallel by adding DMSO or saline preincubated with venom, instead of sponge extracts.

### 4.7. Antiedematogenic Activity

Edema-inducing activity of *B*. *jararaca* (0.7 µg/g) or *L*. *muta* (0.4 µg/g) venom was determined according to [54], with minor modification. Groups of five mice received subcutaneously (s.c.) 50 μL of *B*. *jararaca* venom (7 mg/kg) or *L*. *muta* (7 mg/kg) in the right paw, while the left paw received 50 μL of saline or DMSO. One hour after injection, edema was evaluated as the percentage increase in weight of the right paw compared to the left one. Antiedematogenic activity was performed by incubating the extracts of sponges (140 mg/kg) with *B*. *jararaca* or *L*. *muta* venom for 30 min at room temperature, and then mixture was injected into mice. Control experiments were performed by incubating venoms with DMSO or saline.

### 4.8. Antilethality Activity

Groups of five mice received injection intraperitoneally (i.p.) of *B*. *jararaca* venom (6 µg/g). The antilethality experiment was performed by mixing *B*. *jararaca* venom with the extracts of sponges (14 µg/g) for 30 min at 37 °C, and then mixtures was injected i.p. into mice. Also, *B*. *jararaca* venom was injected i.p into mice, and 15 min later, the sponge extracts were injected i.p. or i.v. Control experiments were performed by incubating venom with DMSO (1%, *v*/*v*, final concentration) or with saline. The volume of the injection was 0.1 mL, and after injection, the number of dead mice was counted in each group.

### 4.9. Statistical Analysis

Results are expressed as means ± SEM obtained with the indicated number of animals or experiments performed. The statistical significance of differences among experimental groups was evaluated using the Student’s *t* test and *p* values of ≤0.05 were considered statistically significant.

## 5. Conclusion

This research demonstrated that marine sponges are undoubtedly rich sources of biologically active-molecules with antivenom potential able to inhibit the main toxic effects of *L*. *muta* and *B*. *jararaca*. Substances derived from sponges could be used as antivenom preparations or as aids to complement other treatments of snakebites. This study also highlights bioprospecting approaches, aimed to explore the rich flora and fauna in the Brazilian oceans in order to enhance the discovery of new antivenom molecules. However, in-depth scientific investigations are imperative to evaluate the antivenom potential of natural products in order to derive therapeutically effective treatments for snakebites. It should be emphasized that experimental models for *in vivo* and *in vitro* studies should be developed to closely reflect the clinical situations of snakebites.
